# Comparison of ultrasound-guided and nerve stimulator-guided interscalene blocks as a sole anesthesia in shoulder arthroscopic rotator cuff repair: A retrospective study

**DOI:** 10.1097/MD.0000000000021684

**Published:** 2020-08-28

**Authors:** Jung A. Lim, Shin Yeung Sung, Ji Hyeon Lee, So Young Lee, Sang Gyu Kwak, Taeha Ryu, Woon Seok Roh

**Affiliations:** a Department of Anesthesiology and Pain Medicine, Catholic University of Daegu School of Medicine; b Daegu Wooridul Spine Hospital; c Department of Medical Statistics, School of Medicine, Catholic University of Daegu, Daegu, Korea.

**Keywords:** arthroscopic surgery, brachial plexus block, nerve block, Rotator cuff injury, shoulder, ultrasound

## Abstract

Ultrasound-guided interscalene block (US-ISB) and nerve stimulator-guided interscalene block (NS-ISB) have both been commonly used for anesthesia in shoulder arthroscopic surgery.

This study aims to compare which method provides surgical block as a sole anesthesia. In this retrospective study, 1158 patients who underwent shoulder arthroscopic rotator cuff tear repair surgery under ISB between October 2002 and March 2018 were classified into either the US-ISB or NS-ISB anesthesia groups. Demographic and anesthetic characteristics and intraoperative medications were analyzed after propensity score matching and compared between the 2 groups.

There was a 0.5% rate of conversion to general anesthesia in the US-ISB group and a 6.7% rate in the NS-ISB group (*P* < .001). The volume of local anesthetics used for ISB was 29.7 ± 8.9 mL in the US-ISB group versus 38.1 ± 4.8 mL in the NS-ISB group (*P* < .001). The intraoperative use of analgesics and sedatives such as fentanyl, midazolam and propofol in combination was significantly lowered in the US-ISB group (*P* < .001).

US-ISB is a more effective and safer approach for providing intense block to NS-ISB because it can decrease the incidence of conversion to general anesthesia and reduce the use of analgesics and sedatives during arthroscopic shoulder surgery.

## 1 Introduction

Both general anesthesia and regional anesthesia or a combination have been previously employed for shoulder surgery. Interscalene block (ISB) is the commonly used regional anesthesia technique, as it can offer complete anesthesia and postoperative pain control for shoulder surgery.^[1–3]^ However, ISB as a sole anesthetic technique for arthroscopic shoulder surgery remains a challenge, as it requires more time and work from anesthesiologists to increase the success rate of block, to keep the “awake” patient comfortable during the operation.

Although the classic Winnie approach (anterior approach) is commonly performed, today most clinicians use nerve stimulator-guided ISB (NS-ISB),^[4]^ ultrasound-guided ISB (US-ISB),^[5–7]^ or a combination^[8]^ to determine the injection point. Since the advent of ultrasound to perform ISB, the superiority of this approach to NS-ISB has been debated.^[5,8,9]^ NS-ISB is known to have a similar efficacy, success rate, and postoperative neurological symptoms compared to US-ISB among experts.^[10]^ However, US-ISB has several potential advantages. The ultrasound allows real-time visualization of anatomical structure and needle tip when performing ISB. Furthermore, ultrasound can confirm whether the size or shape of the nerve is changed while scanning along the path of nerves, and it is also possible to differentiate whether the nerve is normal or pathological by measuring the cross sectional area of the nerve.^[11]^ In addition, the ultrasound is capable of identifying not only the nerve but also musculoskeletal structures surrounding nerves using the same scanning window, enabling to accurately target several desired structures with a single needle injection. Therefore, ultrasound is a useful tool not only to improve the accuracy of diagnosis and anesthesia, but also to enhance the treatment efficacy of diseases such as shoulder impingement syndrome.^[12]^

US-ISB increases the quality of the motor and sensory blockade and decreases incidence of paresthesia and local anesthetic toxicity.^[13]^ Additionally, US-ISB has advantages including fewer needle passes,^[14]^ shorter block performance time,^[15]^ and greater success rate of ISB.^[9]^

Because of these findings, US-ISB may confer greater safety to patients compared to NS-ISB, but comparative studies are limited between the 2 approaches when an ISB, focusing on the intraoperative period, with only 1 surgeon in the same operation for 1 disease, is performed as surgical anesthesia without sedation or general anesthesia. Furthermore, the success of ISB is crucial in terms of surgical anesthesia, but the definition of “block success” or “quality of block” is variable and differs in previous studies.^[16]^

Here, we aim to determine whether US-ISB reduces block failure rate and improves the quality of ISB relative to NS-ISB through comparison of the conversion ratio to general anesthesia and intraoperative use of analgesics and sedatives in arthroscopic rotator cuff tear surgery.

## 2 Methods

**Figure 1:**
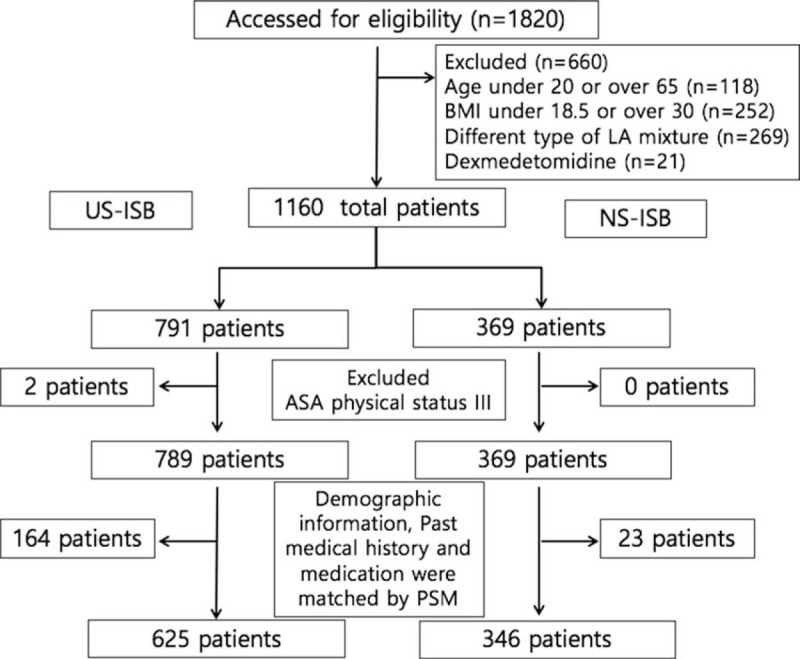
Flow chart of the patient inclusion and exclusion. BMI = body mass index, LA = local anesthetics, NS-ISB = nerve stimulator-guided interscalene block, PSM = propensity score matching, US-ISB = ultrasound-guided interscalene block.

This study retrospectively analyzed data of patients who received arthroscopic rotator cuff repair surgery under an ISB at our hospital between October 2002 and March 2018. Before the study commenced, approval was obtained from the Institutional Review Board of our hospital (CR-19-150), and the study was registered at http://cris.nih.go.kr (KCT0004538). Between these dates, arthroscopic rotator cuff repair surgery under ISB was performed on 1820 patients. Of these, 662 patients were excluded from our study based on the following criteria: adolescents (age below 20 years) or elderly (age above 65 years), body mass index under 18.5 or over 30 kg/m^2^, American Society of Anesthesiologists physical status classification III, different mixture type of local anesthetic such as ropivacaine only, and the use of dexmedetomidine for sedation. Anesthetic records of 1158 patients were reviewed for demographic information, American Society of Anesthesiologists physical status classification, surgical site, and underlying diseases. These parameters were considered confounding variables and were balanced between 2 groups using propensity score matching (Fig. 1). The logit was used for the caliper indicating the maximum width of the caliper for which matching should be done, the value of caliper size was set at 0.2 and matching ratio was 2:1.

To evaluate success rate and adequacy of ISB, the ratio of conversion to general anesthesia and intraoperative administration of analgesics and sedatives was reviewed and compared between the 2 groups from anesthesia records.

Statistical analyses were performed using IBM SPSS software version 25.0 (IBM Corp.,). Cross analysis or Fisher exact test were used for ordinal data, and the independent sample *t* test was used for average comparison of scale data. Descriptive data are presented as numbers (%) or mean ± standard deviation. *P* values of < .01 were considered significant.

Our standard techniques of ISB and intraoperative management for patients undergoing shoulder surgery were as follows. After admission to the operating room, noninvasive arterial blood pressure, pulse oximetry, and electrocardiography were applied. The patient was placed in a supine position with the face directed to the contra-lateral side of surgery, and a nasal cannula of oxygen at 2 L/min was applied. The patient's neck was sterilized using an iodine solution and sterile drapes were applied. All procedures were performed by 5 anesthesiologists with more than 10 years of experience in nerve block.

### 2.1 US-ISB

**Figure 2:**
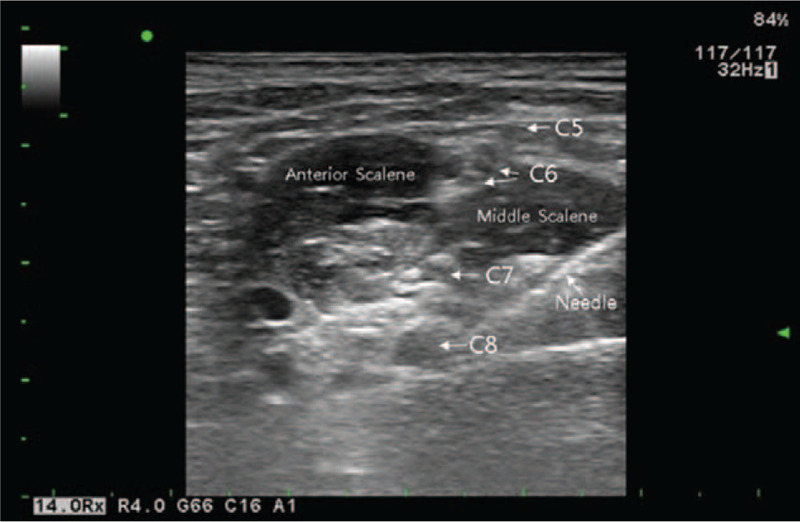
Ultrasound-guided interscalene block. The needle tip is placed between the nerve roots of C7 and C8. C = cervical.

In US-ISB group, the patient's interscalene groove was identified. With a 5 to 13 MHz linear phased array probe of ultrasound (UST-5411, Hitachi Aloka Medical, Ltd) placed parallel to the clavicle and the sonographic beam directed to the first rib, the subclavian artery was confirmed first, as a landmark with the brachial plexus divisions around it. From this position, the brachial plexus nerves were followed in a cephalic direction until the scalene muscles were visualized as surrounding the trunks of the brachial plexus in the interscalene groove. Local anesthesia infiltration was administered as 1 mL of 2% lidocaine in the needle insertion site. A 50 mm, 22-gauge insulated needle (Stimuplex insulated B Braun Medical, Germany) was introduced from lateral to medial, parallel to the interscalene groove using an in-plane technique such that the entire needle was visualized. The brachial plexus was well noted on the cricoid cartilage level, and the probe was aligned to visualize the anterior scalene muscle, cervical (C)5-C6 nerve root, and middle scalene muscle retrospectively. Once the needle tip was seen close to the plexus trunks, the mixture of lidocaine or mepivacaine 1% and ropivacaine 0.25% was injected with frequent aspiration and the spreading pattern of the local anesthetic was visualized using the ultrasound. If any resistance to injection was encountered, or intraneural injection was suspected, the needle tip was repositioned under ultrasound guidance and then local anesthetic was injected (Fig. 2).

### 2.2 NS-ISB

For the NS-ISB group, the interscalene groove was identified at the cricoid cartilage level of trachea and local anesthesia infiltration was administered as 1 mL lidocaine 2% in the needle insertion site. A 50 mm, 22-gauge insulated needle (Stimuplex insulated B Braun Medical, Germany) was introduced from lateral to medial, parallel to the interscalene groove. A 2 Hz, 1 mA stimulus nerve stimulator (Stimuplex, HNS12, B Braun, Germany) was connected to the needle and the needle was inserted with 1 mA stimulus until muscle trigger was noted on the elbow, first, and second fingers. The stimulus was then decreased to 0.3 mA and if muscle trigger was still noted, same type as with US-ISB of local anesthetic was injected in divided doses with frequent aspiration.

After ultrasound or nerve stimulator-guided block placement, the patient was placed in a sitting position for surgery, and sensory block was assessed using an ice pack to ask the patient if the cold sensation in the shoulder was reduced compared to the opposite shoulder. If the block failed, the anesthesiologist changed the anesthesia type from ISB to general anesthesia. When incomplete blocks of ISB occurred or when the patient experienced discomfort or requested sedation, a small dose of fentanyl or midazolam was administrated first. When pain or discomfort could not be controlled by fentanyl and midazolam, these patients received an intravenous bolus of propofol or a propofol infusion. All surgeries were performed by only 1 surgeon.

## 3 Results

After propensity score matching, of 971 patients whose anesthesia records were reviewed, 625 patients were in the US-ISB group and 346 patients were in the NS-ISB group (Fig. 1). The chronological distribution of patients within each group was different (data not shown): whereas most patients in the US-ISB group underwent surgery after 2010, all patients in the NS-ISB group underwent surgery before 2010. Table 1 includes demographic information, which demonstrates that there are no significant demographic differences between the US-ISB and NS-ISB groups except height. Demographic data (Table 1), past medical history, and medication use (Table 2) were not different between the 2 groups.

**Table 1:**
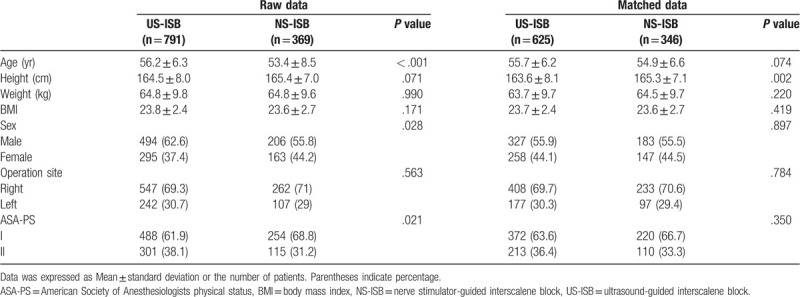
Demographic information.

**Table 2:**
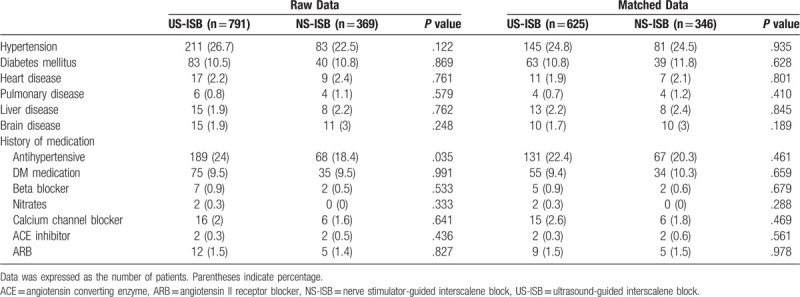
Past medical history and medication.

Three patients (0.5%) in the US-ISB group and 22 patients (6.7%) in the NS-ISB group were converted from ISB to general anesthesia (Table 3). There was no difference between the 2 groups in the time from block to the start of operation (*P* = .436). The total volume of local anesthesia used was significantly lower in the US-ISB group (29.7 ± 8.9 mL) than in the NS-ISB group (38.1 ± 4.8 mL) (*P* < .001) (Table 4). Comparisons of each analgesic and sedative agent administration during operation are presented in Table 5. The use of fentanyl and midazolam was not different between the 2 groups. But both administration of propofol bolus and of propofol infusion were significantly lower (*P* < .001) in the US-ISB group than in the NS-ISB group.

**Table 3:**
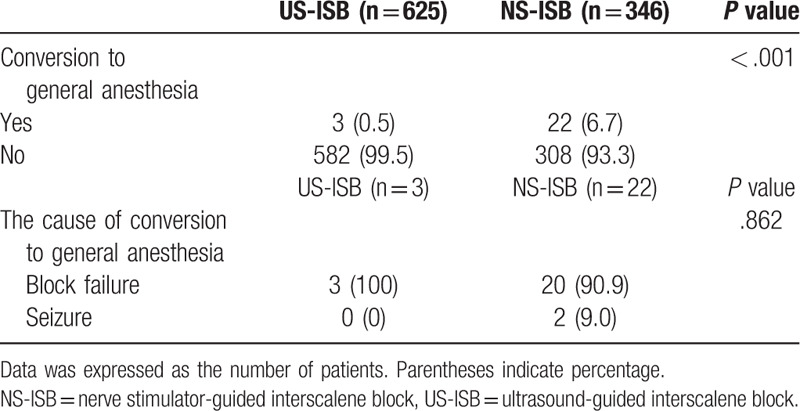
Anesthesia characteristics of patients who were converting from interscalene block to general anesthesia.

**Table 4:**
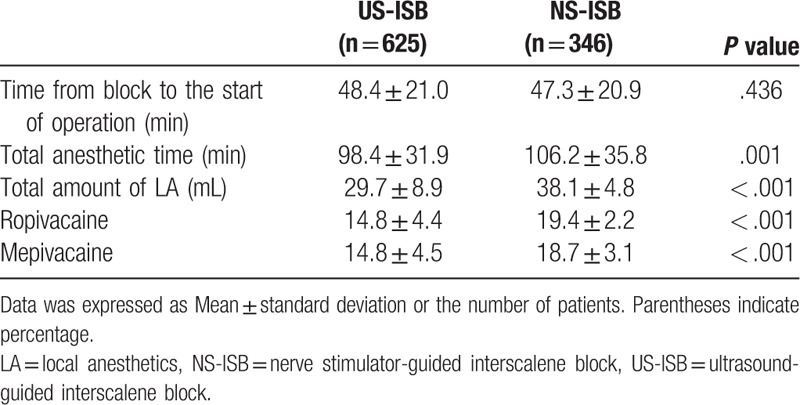
Comparison of anesthesia characteristics between ultrasound-guided and nerve stimulator-guided interscalene block.

**Table 5:**
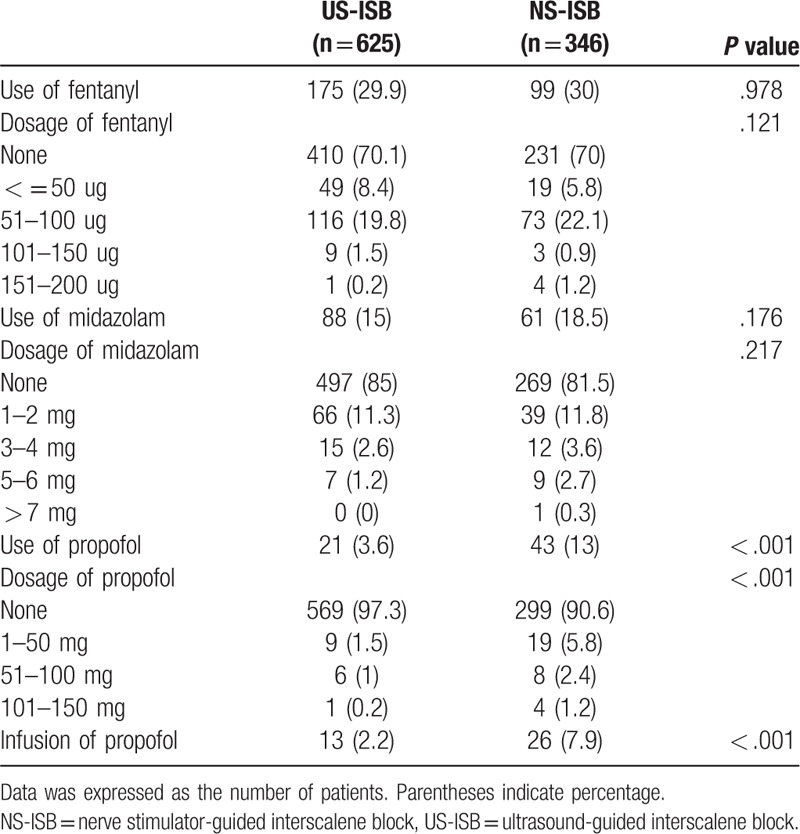
Comparison of administration of intraoperative sedative and analgesic agents between 2 groups.

The combination of intravenous analgesics and sedative agents administered was also less (*P* < .001) in US-ISB group (Table 6).

**Table 6:**
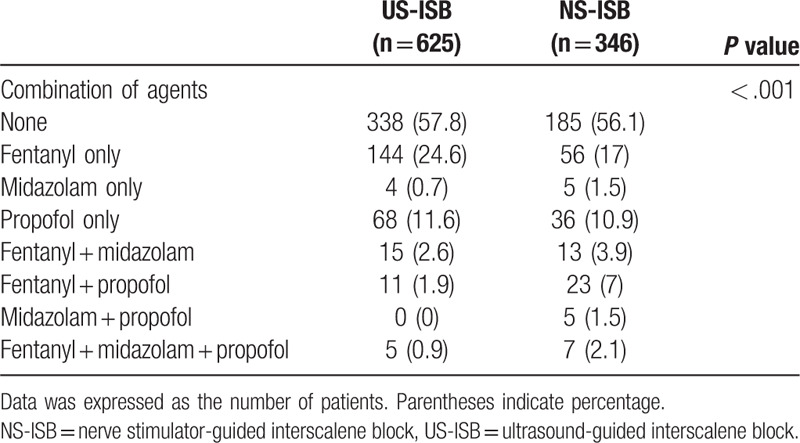
Comparison of the combination of intravenous analgesic and sedative agents.

## 4 Discussion

The purpose of our study was to compare 2 methods when an ISB was performed as a surgical anesthesia without sedation or general anesthesia in patients with rotator cuff tears to determine which method contributes to a more intense block and provides adequate anesthesia during surgery.

US-ISB had a lower incidence of conversion to general anesthesia and a larger reduction of volume of local anesthetics (LA) and intraoperative use of analgesics or sedatives than NS-ISB, indicating that ultrasonography enhances the success rate of block and provides adequate anesthetic maintenance during surgery rather than nerve stimulation.

One previous study suggested that there were no differences in the superiority of NS-ISB and US-ISB for anesthesia administration in shoulder surgery.^[10]^ However, nerve stimulation has several limitations which could explain why NS-ISB was found to be less effective in our study. NS-ISB is an indirect localizing technique and does not guarantee the accurate position of the needle tip. One study showed that the success rate for correct needle-nerve distance (1–4 mm) and local anesthetic spread by nerve stimulation was almost 50%.^[17]^ Also, electrical impedance of the tissue around nerves may affect the motor response to nerve stimulation. The variation of anatomic structure or different arrangement of tissue also affects electric current threshold of nerve stimulation despite an appropriate position of the needle tip.^[18]^ Furthermore, patients with peripheral neuropathies due to underlying disease such as diabetes mellitus may require more nerve stimulator currents, and these risks could cause neurological injury^[19]^ and should thus be considered in light of patient characteristics before using nerve stimulation.

Another study by Kapral et al^[9]^ also reported a higher success rate among US-ISB patients (99%) than NS-ISB patients (91%). The higher success rate of US-ISB could be attributed to several potential advantages of US-ISB. Unlike NS-ISB, US-ISB allows direct visualization of nerves, the needle tip, and the injecting pattern of LA, which allows for greater accuracy. If the distribution of LA is inadequate, US-ISB allows needle repositioning for a more accurate nerve block.

Moreover, ultrasonography makes it possible to find the C8 nerve root that innervates the posterior lesion of the shoulder. This means that US-ISB could be used to block the C8-T1 level, which is shown to provide a more sufficient blockade and reduction of additional local anesthetic infiltration.^[20,21]^ These advantages of ultrasound could explain why US-ISB showed a lower incidence of conversion to general anesthesia than NS-ISB in our study.

The adequacy of anesthesia and patient satisfaction during surgery should be considered in performing ISB as a sole anesthesia. Our study was a retrospective study, which had a limitation of not directly asking all patients for satisfaction, but compared intraoperative analgesics and sedatives alone or combination doses between the 2 groups as an indicator of intraoperative patient satisfaction and appropriateness of anesthesia. Singh^[22]^ found that the block success rate and patient satisfaction were 99.0% with ultrasound-guided ISB, and Weber and Jain^[23]^ reported that the use of narcotics increased with inadequate scalene anesthesia using nerve stimulation. There was a limitation in both studies that results were obtained in a single group without comparison with other groups, but they also accentuated anesthetic precision through the assessment of patient satisfaction and the use of narcotics.

Our institution has a guideline for the administration of intraoperative analgesics and sedatives as described in the methods section, and these would have been performed similarly between the 2 groups. The use of fentanyl and midazolam did not differ significantly between the 2 groups.

Propofol, however, was more frequently used in the nerve stimulator group when administered by bolus or infusion. Furthermore, the dose of propofol was significantly higher in the nerve stimulator group. In addition, the administration of a combination of multiple sedatives and analgesics instead of only 1 kind of agent reflects the insufficiency of anesthesia, which can be demonstrated by the statistically significant higher administration of combination in the NS-ISB group. Therefore, the higher use of intraoperative fentanyl with propofol and midazolam in NS-ISB could be interpreted as low quality of sensory and motor block during shoulder surgery. Elshamaa et al^[24]^ reported that application of US-ISB in shoulder surgery led to lower blood levels of cortisol compared to NS-ISB, which suggests a decreased stress response during surgery and in the postoperative period. Suppression of the stress response in US-ISB reflects the reduction in levels of analgesics supplementation required and thus the higher success rate.

In a prospective controlled trial, Salem^[8]^ compared the conventional ISB using nerve stimulator and a combination method of ultrasound and nerve stimulation. They suggested that there were no differences between patient satisfaction, postoperative pain, and motor power in the 2 groups. However, the technique of US-ISB used by Salem was different from the approach in our study; after sonographic plexus identification and needle fixation, they used nerve stimulation to visualize muscle contractions and then injected local anesthetic in the same way as NS-ISB. In our study, the insulated block needle and not the nerve stimulator was used as routine, such that the nerve stimulator could be connected at any time. In most cases of our study, the “donut” sign was created by injecting local anesthetic around the C5-C8 nerve root under direct visualization using ultrasound. Thus, different techniques to use US-ISB could be a major cause for different results between the 2 studies.

There are several limitations in our study. First, anesthetic records used for this study covered a very long time period, although the type of operation, surgeon, and skill levels of anesthesiologists were same for both groups. As mentioned previously in method, 2 techniques were not used at the same time and there was a difference in the time distribution. The decrease in total anesthetic time in the ultrasound group (Table 4) may reflect that ultrasound helps maintain surgical block properly during operation, but it may also be caused by a surgeon's skill improvement over a long period of time, resulting in a shorter operation time.

Second, US-ISB significantly reduced the local anesthetic volume compared to NS-ISB in our study. Previous reports show that the amount of local anesthetic needed for ISB as the sole anesthetic for shoulder surgery is about 30 to 40 mL, depending on the author.^[6,25]^ Gautier et al reported that successful surgical anesthesia can be achieved with a volume of 5 mL of 0.75% ropivacaine in US-ISB, but a 25% failure rate demonstrated that doses greater than 5 mL may be required for some cases.^[26]^ In this study, NS-ISB generally required 30 to 40 mL total of local anesthetic; however, requirements for total local anesthetic for US-ISB varied from 9 mL to up to 40 mL, depending on the anesthesiologist. The ability of US-ISB to achieve high success rates with very low volumes of local anesthetic may be related to the possibility of intraneural block,^[27]^ but further study is needed to determine the minimum local anesthetic volume required when US-ISB is the sole anesthetic for arthroscopic shoulder surgery.

Finally, US-ISB has been reported to lead to fewer complications such as vessel perforation or nerve injury in periphery nerve block for upper or lower extremity operation.^[28]^ In our study, none of the patients had seizure in the US-ISB group. Two patients in the NS-ISB group presented with seizure, which is believed to have been caused by intravascular injections of a local anesthetic. Further, our posterior approach to performing an US-ISB can reduce damage to the phrenic nerve, which may occur with the anterior approach, but the risk of dorsal scapular nerve or long thoracic (T) nerve injury is inevitable. Since these 2 nerves are very small branches and especially the long T nerve emerges almost vertically from the middle scalene muscle, it can be helpful to identify long T nerve by swapping the transducer's posterior edge during scanning and obliquely positioning the transducer.^[29]^ However, detailed information on the complications of ISB such as phrenic nerve palsy and winged scapula was not available in this study due to the limitations of a retrospective study focusing on intraoperative complication, although the complication incidence was very low in both groups. Therefore, we could not determine whether US-ISB reduces complications.

In summary, US-ISB as an anesthetic approach is advantageous over NS-ISB for shoulder arthroscopic rotator cuff repair, as our study found that US-ISB cases had lower incidence of conversion to general anesthesia than NS-ISB to block patients. Further, US-ISB exhibited reduced use of analgesics and sedatives during arthroscopic surgery. Thus, the use of ultrasound in ISB should be more increased in shoulder arthroscopic surgery to improve the surgical anesthesia.

## Author contributions

**Conceptualization:** Woonseok Roh.

**Data curation:** Shin Yeung Sung, Ji Hyeon Lee, So Young Lee.

**Formal analysis:** Sang Gyu Kwak.

**Methodology:** Taeha Ryu.

**Supervision:** Woonseok Roh.

**Writing – original draft:** Jung A Lim, Shin Yeung Sung, Woonseok Roh.

**Writing – review & editing:** Jung A Lim, Taeha Ryu, Woonseok Roh.
